# Radiological artificial intelligence - predicting personalized immunotherapy outcomes in lung cancer

**DOI:** 10.1038/s41698-023-00473-x

**Published:** 2023-11-21

**Authors:** Laila C. Roisman, Waleed Kian, Alaa Anoze, Vered Fuchs, Maria Spector, Roee Steiner, Levi Kassel, Gilad Rechnitzer, Iris Fried, Nir Peled, Naama R. Bogot

**Affiliations:** 1 The Hebrew University, Helmsley Cancer Center, Shaare Zedek Medical Center, Jerusalem, Israel; 2 Ben-Gurion University of the Negev, Be’er Sheva, Israel; 3 The Institute of Oncology, Assuta Ashdod, Ashdod, Israel; 4 The Department of Radiology, Shaare Zedek Medical Center, Jerusalem, Israel; 5 The Institute for Nuclear Medicine, Shaare Zedek Medical Center, Jerusalem, Israel

**Keywords:** Predictive markers, Lung cancer

## Abstract

Personalized medicine has revolutionized approaches to treatment in the field of lung cancer by enabling therapies to be specific to each patient. However, physicians encounter an immense number of challenges in providing the optimal treatment regimen for the individual given the sheer complexity of clinical aspects such as tumor molecular profile, tumor microenvironment, expected adverse events, acquired or inherent resistance mechanisms, the development of brain metastases, the limited availability of biomarkers and the choice of combination therapy. The integration of innovative next-generation technologies such as deep learning—a subset of machine learning—and radiomics has the potential to transform the field by supporting clinical decision making in cancer treatment and the delivery of precision therapies while integrating numerous clinical considerations. In this review, we present a brief explanation of the available technologies, the benefits of using these technologies in predicting immunotherapy response in lung cancer, and the expected future challenges in the context of precision medicine.

## The challenge of personalizing treatment

In oncological practice, personalized medicine—which has traditionally relied on the molecular characterization of tumors using genomic, and proteomic techniques, aims to customize treatment and ultimately guide clinical decisions and therapeutic interventions^1^. However, this approach has presented substantial difficulties for clinical oncologists, who are expected to develop a precise treatment plan for each patient based on these complex clinical data.

Personalized medicine in oncology traditionally involves invasive procedures to gain access to cancerous tissues, which only provides insight into small parts of the tumor and occasionally may be complicated by tumor placement and the potential risks of surgical biopsies^2–4^. While ‘liquid’ biopsies are a safer alternative to procuring solid tumor tissue and have shown promise in personalized medicine owing to the presence of circulating tumor DNA/RNA^5–8^, both liquid and tissue biopsies capture only a small spatiotemporal snapshot of notably heterogeneous and constantly evolving solid tumors^9^. Thus, tumor heterogeneity presents a barrier to personalized treatment^10^

AI-based analyses of radiological features from standard-of-care (SOC) images can serve as biomarkers and could play a considerable role in overcoming the challenges of personalized medicine. Although there are currently limitations to the technology, particularly with smaller tumors, the next-generation imaging analysis could ultimately be used to predict responses to therapies and develop more precise treatment plans for individual cancer patients in the era of personalized medicine. More recently, efforts to standardize radiomics via the integration of AI algorithms have steered the field of radiomics away from handcrafted features, thereby reducing bias and increasing accuracy and generalizability^11–16^. This will only serve to increase the diagnostic and prognostic accuracy of radiomics and strengthen associations between extracted data and biological and clinical endpoints to expedite and further optimize personalized treatment.

## Why next-generation medical imaging analysis?

Diagnostic imaging—a qualitative and non-invasive method for assessing internal structures—has historically been the cornerstone of analytical and diagnostic workflows in oncology and helps to inform the course of treatment in clinical oncology practice^10,17^. While routine medical imaging (i.e., ultrasound [US], computed tomography [CT], magnetic resonance imaging [MRI], and positron emission tomography [PET]) can capture the overall spatial and temporal heterogeneity of solid tumors, qualitative visual assessments lack the granularity and objectivity to assess inter- and intratumour heterogeneity within the complex tumor microenvironment (TME)^9^.

Solid tumors not only vary in size and shape over time but are also known to be both phenotypically and genotypically heterogeneous, where tumor cells can vary in cell type, genomic sequence, gene expression, vascularization, oxygenation, metastatic potential, and response to treatment within the TME^18–24^. Furthermore, it has been established that heterogeneous tumor features are valuable prognostic indicators of variability in tumorigenesis, treatment efficacy, metastatic potential, and patient outcomes^9,10,25–28^.

Radiomics is an evolving field of study in which large numbers of quantitative features are extracted from standard-of-care (SOC) radiographic images and linked to clinical outcomes using either handcrafted or machine learning methodologies^29^. First proposed in 2012, radiomics leverages the heterogeneous nature of solid tumors by converting medical images into “mineable data” via the high-throughput extraction of high-dimensional quantitative features^30–32^. Burgeoned by increasing computational capabilities, radiomics has been used for nearly a decade to link quantitative features of tumors (i.e., intensity, texture, shape, volume, and wavelets) to clinical endpoints, such as response to therapy, metastases, and survival in a variety of cancers using multiple imaging modalities^3,12,16,33–35^. Despite the utility of this approach^3,36^, issues such as inconsistencies in image acquisition; motion artifacts; variability in segmentation and image processing; the limited reproducibility of features across centers, studies, and software tools; and analytical variability have been barriers to the adoption of radiomics into clinical workflows (please refer to the Supplemental Material for more technical details)^9,14,37,38^.

Deep learning—a subset of machine learning that uses multilayered artificial neural networks to transform input data (e.g., images) to output data (i.e., diagnostic parameters) while learning increasingly higher-level features in each layer—has been used to automate and optimize the extraction of features from medical images for nearly a decade^39,40^. The integration of DL into radiomics workflows (i.e., deep radiomics) bridges a gap in the field and mitigates many of the prevailing inconsistencies with “handcrafted” radiomics, in which human variability can introduce error at any point in the radiomics pipeline^12,14,38^. Notably, although radiomics and deep radiomics have been well investigated in a variety of cancers and have been used successfully to assess treatment response and predict survival in patients enrolled in clinical trials^41^, to the best of our knowledge, radiomics and deep radiomics have not yet been utilized in real-world clinical workflows.

Thus, while the integration of radiomics and AI offers oncologists a unique opportunity to predict personalized responses to immunotherapy in lung cancer prior to treatment, there are some barriers to incorporating these techniques into real-world clinical scenarios. The present paper aims to briefly review the current application of these technologies in lung cancer, as well as current obstacles to integrating radiomics techniques into clinical lung cancer workflows (please refer to the Supplemental Material for more technical details).

## Clinical applications

**Fig. 1:**
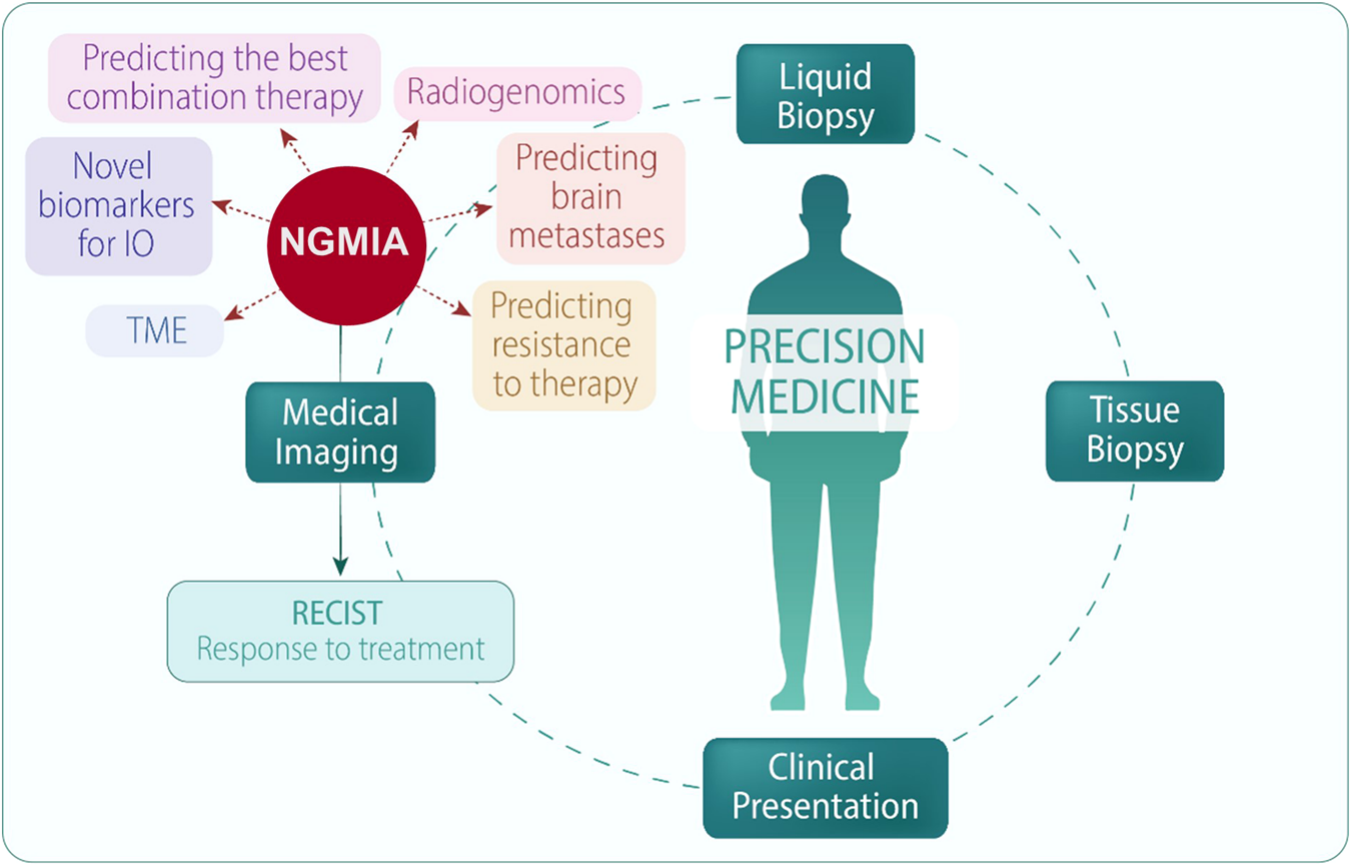
Clinical Opportunities in Precision Medicine via the integration of next-generation medical imaging analyses (NGMIA). Currently, medical images play a predominant role in evaluating treatment responses. However, the introduction of innovative strategies such as radiomics and artificial intelligence provides a domain of predictive capabilities, augmenting treatment decision-making and complementing traditional tissue and liquid biopsy approaches.

There is an indisputable link between the radiomic features of tumor heterogeneity—both handcrafted and AI-enabled—and biological and clinical end points. In lung cancer, radiomic biomarkers have been shown to predict distant metastases^42–44^, malignancy in pulmonary nodules^45,46^, primary tumor stage^10^, histology^10,47^, pathological response after chemoradiation^48^, disease recurrence^49^, somatic mutations^50–54^, gene expression profiles and molecular pathways^10,55^, adverse events^56^, and survival^54,57–60^. Likewise, radiomic signatures can also be used to optimize treatment plans in NSCLC patients, such as chemoradiotherapy^61^, immunotherapy^34,54,62^, and tyrosine kinase and immune checkpoint inhibitors^54,63^. In the following sections, we will summarize the potential roles of handcrafted and deep radiomics in predicting personalized responses to immunotherapy in lung cancer (Fig. 1).

### Biomarkers for response to immunotherapy: beyond PD-L1

Immune checkpoint inhibitors that target biological biomarkers such as programmed death ligand 1 (PD-L1) have revolutionized lung cancer treatment as an alternative to cytotoxic chemotherapies^64,65^. However, despite the success of immunotherapy for some patients, a substantial number of patients do not experience clinical benefit, even in highly selected cohorts^65,66^. In this section, we will explore how the integration of radiomic biomarkers with SOC biomarkers could substantially impact patient care by helping to predict the response to immunotherapy.

Delta radiomic (DelRadX) features—changes in radiomic features over time—have been used to predict clinical outcomes in a variety of cancers^67^. A DelRadX signature based on CT images at baseline and at the end of the second treatment cycle performed satisfactorily in distinguishing between responders and nonresponders when used in combination with the clinical factor of distant metastasis (area under the curve [AUC] of 0.83 vs 0.81, respectively)^34^. Furthermore, this DelRadX signature was significantly more predictive of the response to immunotherapy than PD-L1 expression alone (*p* < 0.001). Khorrami et al.^68^ found that DelRadX using intranodular and perinodular texture features of malignant NSCLC nodules from CT images predicted the response to immunotherapy and overall survival (OS). Sun et al.^69^ determined the radiomic signature of CD8 cells using RNA-seq data combined with a radiomic analysis of solid tumors in a variety of cancers (including lung cancer) and validated it using two independent cohorts of patients. The radiomic signature of CD8 cells was predictive of the immune phenotype (dense vs low CD8 cell infiltration). In this study, a higher radiomics score—derived from five radiomic features extracted from each lesion, two discrete labels related to lesion location, and one imaging acquisition-related variable—was significantly associated with the response to anti-PD-1 or anti-PD-L1 monotherapy at both 3 and 6 months after the start of treatment, as well as OS.

Additionally, Trebeschi et al.^70^ developed a radiomics biomarker from CT imaging by training a model on all lesions (i.e., progressive, stable, and responding) to discern progressive disease. This biomarker was significantly associated with response vs nonresponse to immunotherapy in NSCLC patients. More specifically, tumors with increased morphological heterogeneity, nonuniform density patterns, and compact borders were more likely to respond to immunotherapy, while more compact and spherical profiles were associated with better response in nonresponding tumors. A gene enrichment analysis was performed to define the biological basis of the radiomic biomarker and found significant associations with pathways involved in mitosis, indicating a relationship between increased cell division and response to immunotherapy. Several other studies have used radiomic features to stratify NSCLC patients and to significantly predict survival outcomes in patients treated with immunotherapy^71,72^. Furthermore, other studies demonstrate that prognostic biomarkers perform best when combining radiomic, genetic, and clinical data, highlighting the complementary nature of these analyses^55^.

Tumor mutational burden (TMB) has also been shown to be a significant predictor of immunotherapy efficacy^73^. The deep learning model 3D-DenseNet was used to estimate the target tumor area in CT images from 327 NSCLC patients with TMB data and identified 1020 deep features to distinguish between patients with high and low TMBs. The TMB radiomic biomarker was a significant predictor of immunotherapy efficiency and distinguished between high-TMB and low-TMB patients in the training and test cohorts better than histological subtype. Moreover, the TMB radiomic marker was more robust than both the radiomic and clinical models.

Last, as somatic mutations, such as *EGFR*, are known to be associated with low response rates to anti-PD-1/PD-L1 immunotherapy^74^, DL models that predict mutation status from imaging could also predict response to immunotherapy. A study that used a PET/CT-based DL model with high accuracy in predicting *EGFR* mutation status across patient cohorts demonstrated a significant association between high *EGFR* mutation status and low durable clinical benefit, low PD-L1, high hyperprogression, and lower progression-free survival (PFS) in immunotherapy patients, indicating that *EGFR* mutation status assessed using SOC imaging could indeed serve as a biomarker to predict response to immunotherapy^54^.

### Predicting the best combination therapy

Owing to the heterogeneous nature of lung cancer and the substantial number of patients who do not experience clinical benefit from immunotherapy alone, combining therapies can be an effective way to improve outcomes^75,76^. In this subsection, we will discuss the use of radiomics and DL technologies in supporting treatment decision making and predicting the best combination therapy to suit each patient.

A study by Sun et al.^77^ used a CD8 T-cell-associated radiomics signature to predict lesion response in irradiated and abscopal lesions using clinical data from patients with advanced solid tumors (including lung) from six independent clinical studies of combined radiotherapy and immunotherapy. The authors found that CD8 radiomics scores exhibited significantly higher tumor responses (i.e., decrease in lesion size) and that more heterogeneous CD8 radiomics scores across lesions were associated with mixed response or uniform progression, poor PFS, and OS. Additionally, heterogeneous CD8 radiomics scores based on the entropy of the distribution were significantly associated with the response evaluation criteria in solid tumors (RECIST)-based response in abscopal tumors. Not only can this study help to inform prognostic features for combined therapy and inform the choice of target lesion, but this study demonstrates that a radiomics score previously validated in a cohort treated with immunotherapy alone could be predictive in the context of combined therapies^69^.

Another study that used radiomics to study the response to combined therapies showed that a radiomic risk score (RRS), calculated using radiomic textural patterns within and around NSCLC nodules from pretreatment CT images, was found to be significantly associated with PFS and OS (*p* < 0.05) in patients treated with both chemoradiation and chemoradiation + immunotherapy^78^. The RRS also effectively stratified between low and high risk and was significantly associated with OS in the low PD-L1 group.

### Predicting resistance to therapy

Acquired and inherent resistance mechanisms continue to be a considerable factor in poor lung cancer prognosis^79^. Most patients with NSCLC develop primary resistance during PD-1/PD-L1 monotherapy, of which only 15–20% exhibit a partial or complete response^80^. Acquired resistance can also occur despite initial clinical benefits. Notably, there are numerous mechanisms of resistance to immunotherapy in NSCLC beyond PD-L1 expression that could serve as predictive radiomic biomarkers in precision therapy, such as high microsatellite instability/defective DNA mismatch repair; tumor mutational burden; DNA polymerase (POLE) mutations; cytokine expression (e.g., interferon-gamma [*IFN-γ*], tumor necrosis factor-alpha [*TNF-α*], and interleukins); and point mutations, deletions, or homozygous or heterozygous loss of beta-2-microglobulin (*B2M*)^81–84^. Similarly, molecular mechanisms and tumor characteristics such as the overexpression of oncogenes (e.g., *MDM2*^85^), *EGFR* mutations and associated changes in the TME^86^, or tumor hypoxia^87^ can also be predictive of the response to immunotherapy.

While there is a growing body of research that links radiomic features of tumors and the TME to tumor genotype, histology, immune state and clinical end points, studies that investigate radiomic biomarkers of resistance — both handcrafted and AI-enabled — are sorely lacking. The use of DelRadX to elucidate genotypic and phenotypic changes in response to treatment over time could expose underlying mechanisms of resistance. Public datasets for gene expression^88^ in NSCLC could be leveraged to identify prognostic biomarkers or to assess the multivariate performance of radiomic signatures^10^. Furthermore, existing knowledge of somatic mutations involved in resistance to targeted therapies^74^ or the role of immune cells (i.e., CD8 T cells) in tumor growth, metastasis, and resistance to immunotherapy^89^ could be leveraged to develop radiomic signatures that predict resistance to therapy.

### Predicting side effects

The early detection of treatment-related adverse events is crucial for improving patient outcomes. Radiomics can also be applied to predictions of life-threatening adverse events such as cardio-toxicity, pneumonitis, and hyperprogression, as well as the misinterpretation of pseudo progression, to optimize patient care.

Several studies suggest that PET and CT-based radiomic features in NSCLC patients undergoing immunotherapy could be used to predict inflammatory conditions such as immunotherapy-induced pneumonitis^90,91^ or risk for developing severe immune-related adverse events (irSAEs)^92^. By capturing features at baseline that are predictive of potential irSAEs, treatment plans could be optimized early to minimize risk. For example, Mu et al.^92^ found that creating a radiomics nomogram that included a radiomics score based on features extracted from baseline (i.e., pretreatment), the type of immunotherapy, and dosing schedule effectively predicted patients with and without irSAEs with AUCs of 0.92, and 0.88 in the testing, and validation sets, respectively. However, the sample size of this study was relatively small for positive cases. As rule-of-thumb guides for binary classification studies suggest that sample size should be 10–15 times that of the number of features used, studies with smaller samples sizes should be interpreted with caution^93^.

Biomarkers that differentiate between pseudoprogression, progression, and hyperprogression during immunotherapy are lacking. Hyperprogression is an adverse reaction to anti-PD-1/PD-L1 immunotherapy in some NSCLC patients and is associated with significantly shorter survival. Vaidya et al.^94^ extracted 198 intratumoural and peritumoural radiomic textural patterns and tortuosity features of the nodule-associated vasculature from pretreatment CT scans. They found that the top features associated with hyperprogression were able to distinguish between hyperprogression and other patterns with AUCs of 0.85 in the training set and 0.96 in the validation set. A study by Tunali et al.^95^ created rapid disease progression phenotypes composed of time to progression (TTP)/tumor growth rates and hyperprogression in NSCLC patients being treated with immunotherapy using the following baseline predictors: patient demographics, clinical data, driver mutations, hematology data, and radiomic features from CT scans. As a result, the authors identified several effective clinical-radiomic models that predicted rapid disease phenotypes with AUC values of 0.80–0.86 and classified patients with TTP of <2 months and hyperprogression with 73 and 82% accuracy, respectively. Finally, DL biomarkers for somatic mutations such as *EGFR* have also been shown to be associated with high hyperprogression in patients undergoing immunotherapy^54^ and thus show promise as noninvasive biomarkers for predicting adverse events.

### Tumor microenvironment

The tumor microenvironment (TME) is known to promote the growth and metastasis of lung tumors and has recently gained recognition as an important factor in understanding tumor behavior and response to immunotherapy^96^. Here, we will discuss the role of radiomics and DL in elucidating TME characteristics related to treatment outcomes.

Peritumoral radiomics—the use of radiomic techniques to assess heterogeneity of the peritumoral environment—has been investigated in a wide variety of cancers, in which the inclusion of the peritumoral region increases the predictive power of radiomic signatures compared to intratumoral signatures alone in a variety of cancers (see ref. ^16^ for review). Features of the TME have been specifically shown to have significant predictive and prognostic value in lung cancer^68,94^. For example, radiomic features of the tumor rim (i.e., 3 mm outside the tumor border) have been shown to be predictive of distant metastases in NSCLC, where the combination of clinical data and rim signatures was the most effective for stratifying patients^97^. Likewise, Hosny et al.^60^ used DL to create prognostic signatures of quantitative imaging features and found that the tumor–stroma interfaces exhibited the largest contributions to the prognostic signature, highlighting the importance of the TME in patient stratification. Furthermore, the authors created activation heatmaps overlaid on CT images to visualize the “importance” of each node or voxel relative to the final prediction, both within and beyond the tumor. A subsequent analysis that disregarded the data beyond the tumor resulted in a substantial drop in prognostic power, thereby confirming the importance of textural features in the tumor-surrounding region. A radiomics model developed by Tang et al.^98^ characterized the immune state of the TME in NSCLC patients using baseline CT images, percent tumor PD-L1 expression, and the density of tumor-infiltrating lymphocytes (CD3) to stratify patients into four clusters that were significantly correlated with overall survival (OS)^82^. The most favorable outcome group was characterized by low CT intensity and high heterogeneity, low PDL1, and high CD3 infiltration, suggesting a high immune-activated state. Finally, the infiltration of CD4 and CD8 T cells is also known to be an important mediator of responses to immune checkpoint inhibition^96^ and could make effective targets as biomarkers.

### Radiogenomics

Radiogenomics is the study of the connections between SOC radiographic images and tumor genomics. While tumor molecular profiling is becoming SOC for NSCLC and provides a vast amount of information for personalized treatment, traditional molecular biomarker analyses often fail to capture the full picture of spatial and temporal intratumoural heterogeneity. Handcrafted and deep radiomics can bridge the gap between imaging phenotypes and tumor genomics to identify noninvasive, image-based genetic biomarkers to elucidate the underlying mechanisms of resistance and response to therapy and ultimately improve patient care.

Tumor genotype plays an important role in personalized treatment for lung cancer patients, where mutations in common proto-oncogenes and oncogenes such as *EGFR*, *ALK*, *ROS1*, and *RET* have been associated with radiomic signatures ^see 11 for review^. In particular, *EGFR* has been widely investigated as a noninvasive biomarker using both handcrafted^50,53^ and deep radiomic methods^52,54,59^. Aerts et al.^10^ found that radiomic data were strongly prognostic and were associated with underlying patterns of gene expression in a lung cancer dataset using a gene-set enrichment analysis (GSEA) of 21,766 genes. Notably, features III and IV of the four-feature radiomic signature were strongly correlated with cell cycling pathways. Grossman et al.^55^ linked numerous imaging features based on intratumoural heterogeneity to RNA polymerase expression, the autodegration pathway E3 ubiquitin ligase COP1, p53, cell cycle regulation checkpoints, TGF-β signaling, mitochondrial pathways, lipoprotein metabolism, TRAF6-mediated NFkB activation, and axon guidance. Furthermore, several imaging features were linked to *EGFR*, *KRAS*, and *TP53* mutants, as confirmed by Sanger sequencing. Another study used a 3D-CNN and transfer learning approach to identify both prognostic signatures using NSCLC CT images and correlations between the radiographic phenotypes quantified by CNN and global gene expression patterns using a pre-ranked GSEA^60^. Similar to other radiogenomic analyses in NSCLC, the authors found that the most significantly enriched pathways were linked to the cell cycle and transcriptional processes.

### Predicting brain metastases

Lung cancer is one of the most common primary tumors leading to brain metastases (BrMs), accounting for more than 50% of all brain tumors^99^. The presence of BrMs plays a major role in guiding treatment, as brain-penetrating therapies are generally necessary in combination with immunotherapy to improve sensitivity to treatment. Radiomics has been used extensively to predict the local response for BrMs after stereotactic radiosurgery^100^, differentiate between BrMs and glioblastoma^101^, predict the primary tumor of origin^102^, assess the diagnostic ability of BrMs to predict *EGFR* mutation status in primary lung cancer BrMs^103^, and predict survival^104^. While the risk of BrMs in NSCLC patients has been predicted using a nonradiomic nomogram^105^ and based on total lesion glycolysis and metabolic tumor volume^106^, very few studies have used radiomics or DL to predict the development of brain metastases. To the best of our knowledge, no studies have leveraged DL to this end.

One study found that a radiomics score—based on seven potential predictors of BrMs in curatively resected locally advanced NSCLC patients—was significantly associated with BrMs in both the training and validation cohorts, in which combining clinical risk factors and radiomics data improved performance^107^. Additionally, patient smoking status and histology were both independent predictors of BrMs. In contrast, another study found that CT-based radiomics features of primary NSCLC did not improve a model based on clinical data^108^. Nonradiomic prognostic biomarkers of BrMs have been developed by characterizing the functional gene expression signatures of lung tumor tissue, BrMs, and their respective TMEs, indicating that the immune and fibrosis status of BrMs should guide therapeutic strategies^109^. The TMEs of both lung tumors and BrMs were enriched for genes associated with cancer-associated fibroblasts and the extracellular matrix and endothelium, suggesting that fibrosis and angiogenesis play a key role in tumor progression and metastasis. The expression of tumor proliferation genes was higher in the primary tumor. The authors also noted significantly higher expression of epithelial to mesenchymal transition (EMT) genes (i.e., laminins, integrins, and inflammatory and neutrophil-acting chemokines) in both tumors than in the TMEs, as EMT is thought to be the most common mechanism for metastasis^110^. Assessing differentially expressed genes from lung tumor cores between slow and fast metastatic cohorts revealed a metastatic signature gene set that also individually predicted survival in both the study cohort and a lung adenocarcinoma cohort (*n* = 501). These molecular biomarkers could potentially be incorporated into radiomics and deep learning models to predict BrMs in lung cancer patients.

## Future challenges and implementation

While new technologies such as radiomics and DL have substantially advanced the field of personalized immunotherapy treatment in lung cancer, integrating these technologies into clinical workflows will presumably be an uphill climb. Several challenges will need to be addressed before we can create a roadmap to clinical application.

While numerous studies have improved the quality and interpretation of radiomics studies^3^, have worked to standardize radiomic features^37^, and have devised methods of moderating ‘center effect’ to reduce multi-center variability^111,112^, there are still multiple points in the handcrafted radiomics pipeline where errors and variability can be introduced^37^. While DL methods do mitigate issues with handcrafted radiomics—such as time-consuming manual feature selection and inter-observer and intra-observer variability—by minimizing operator input^31,113^, working with DL algorithms is a specialized skill and may not be readily available to radiologists and clinicians. To this end, public dataset repositories such as RIDER^10,60^ and The Cancer Imaging Archive^114^ can be leveraged to validate the performance of radiomic signatures, while open-source toolkits—such as PyRadiomics^115^, RaCaT^116^, and ImaGene^117^—allow researchers to use radiomics without having to develop feature pipelines from scratch^16,118^. Additionally, “how to” guides for implementing radiomics in medical imaging^118,119^ that include tools for both handcrafted and deep radiomics provide a valuable roadmap for those starting out. The use of AI-based tools such as I^3^Lung—which uses biological, molecular, radiological, and clinical data from more than 2000 NSCLC patients to predict individual responses to immunotherapy—could be a straightforward and cost-effective alternative to developing de novo DL models^120^. Lastly, using a transfer learning paradigm on pretrained DL models trained on large datasets makes DL more accessible while mitigating the need for large datasets in clinical workflows (see Supplemental Material for more detail).

Koçak et al.^118^ outlined three-tiered suggestions for radiologists interested in using radiomics: (i) paid software programs, (ii) free programs [see paper for suggestions] that allow radiomic feature extraction using a graphical user interface, or (iii) the development of coding skills necessary to use MATLAB or Python platforms. To become involved in AI, the authors offer a similar three-tiered approach: (i) become part of a data science collaboration, (ii) acquire statistical skills necessary to perform AI tasks without code, and (iii) learn coding language such as Python. Bera et al.^16^ Note that the pathway to regulatory approval is the main roadblock to adopting AI-based predictive and prognostic tools into clinical workflows. Furthermore, the authors state that while billing and reimbursement present yet another challenge, pursuing a regulatory pathway for lab-based diagnostic tests could be a viable option. It is also possible that efforts to unpack the “black box” of AI will increase transparency and explainability on the road to approval (see Supplemental Material for more detail).

No two cancers are alike. Deep radiomics holds the promise of predicting personalized responses to immunotherapy in lung cancer patients using SOC images, thereby expediting treatment and tailoring treatment to individuals. Although the road to adoption could be long, precision oncology will undoubtedly benefit from more objective and accurate characterizations and predictions of disease, ultimately serving to improve patient outcomes.

### Supplementary information

Supplementary

## Data Availability

All data is available by contacting the corresponding author LCR.
