# Supplementary material for: Radiological artificial intelligence - predicting personalized immunotherapy outcomes in lung cancer
Source: NPJ Precis Oncol. 2023 Nov 21;7:125. doi: 10.1038/s41698-023-00473-x (PMC10663598; doi:10.1038/s41698-023-00473-x)
Supplement: Supplementary file 1 — Supplementary [file 41698_2023_473_MOESM1_ESM.pdf]

Supplementary Information

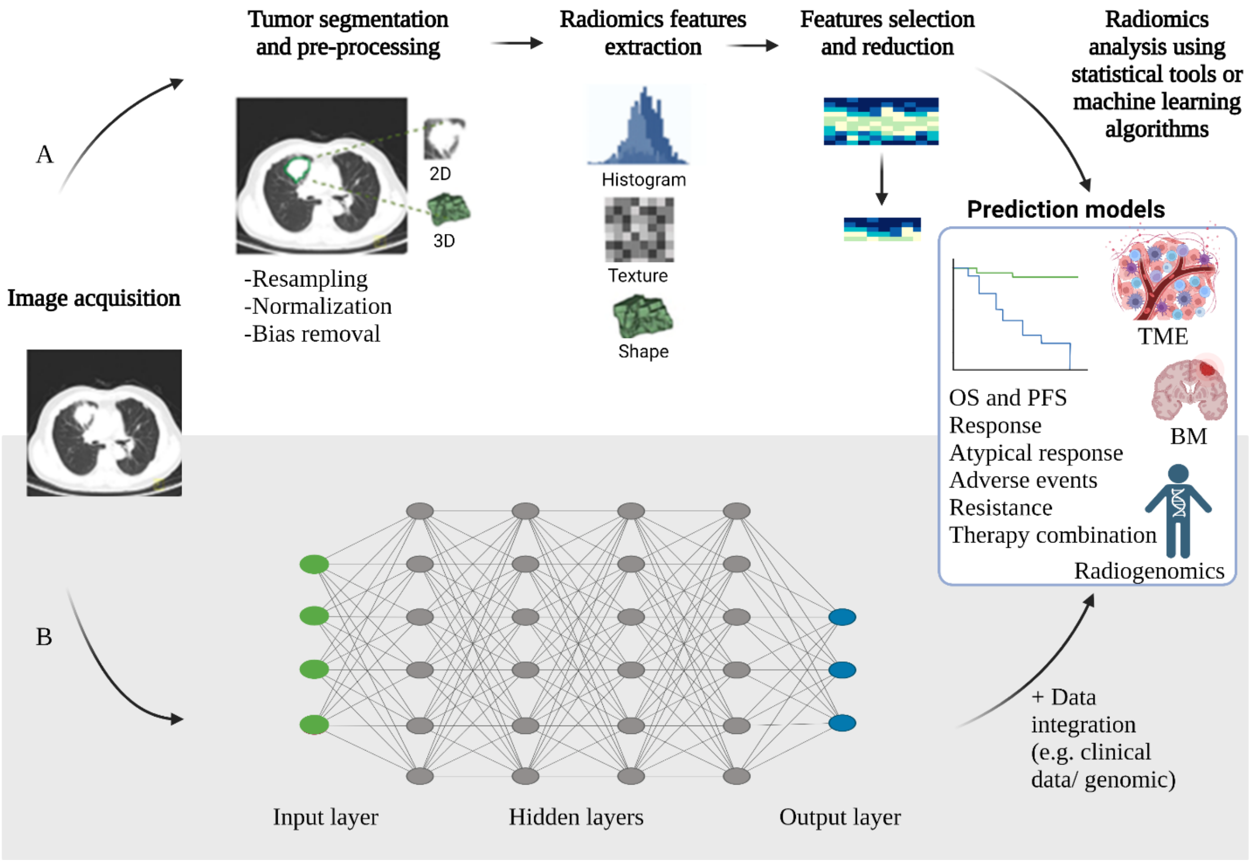

Supplementary Figure 1: Overview of the radiomics pipeline. **(A)** Standard radiomics: the pipeline consists of tumor segmentation, image preprocessing, following features of different categories (e.g., intensity-, texture-, or shape-based), are extracted from regions of interest (ROIs). Extracted features are reduced and important features are outlined in the feature selection process. The final selection feature set is used for classification and model building. **(B)** Radiomic-based deep learning models: images are used directly without labeling ROIs. The three distinct steps of the radiomic workflow (i.e., feature extraction, selection, and classification) can be performed by the same complex algorithm. OS, overall survival; PFS, progression-free survival; TME, tumor microenvironment; BM, brain metastases.

How do radiomics technologies work?

As lung cancer is the second leading cause of cancer-related deaths worldwide[1], early detection, diagnosis, and interventions are paramount to patient outcomes[2]. A thorough understanding of the current radiomics landscape will pave the way for the adoption of these technologies into lung pathology workflows and clinical decision support systems. In this section, we will review how radiomics and deep radiomics work in a basic and understandable manner and will further assess the fusion of these techniques and advanced options for data integration, with particular attention to the use of these technologies in NSCLC.

Radiomics

Radiomics has been referred to as "the bridge between medical imaging and personalized medicine"[3], and has been used to predict numerous biological and clinical endpoints with a high degree of accuracy in a variety of cancers[4,5]. This technology harnesses the heterogeneous nature of tumours by defining and extracting a high number of quantitative 'features' from standard-of-care (SOC) radiographic images, which are converted into mineable data that can be linked to biological and clinical endpoints for clinical decision support[6,7]. Radiomics can be performed on whole tumours, subregions of tumours, tumour-associated vasculature, metastatic lesions, the TME, and even normal tissues[6,8]. While the number and delineation of steps in the radiomics pipeline can vary between studies, there are generally considered to be five steps in the traditional, handcrafted radiomics pipeline: i) image acquisition from SOC images, ii) region of interest (ROI) selection and segmentation (i.e., identifying volumes with prognostic value or the volume of interest [VOI]), iii) extraction of image-based features from the VOI (i.e., texture, shape, histogram, wavelet), iv) data analysis (i.e., training and or discovery of a model using machine learning [ML] or traditional biostatistics) to predict the dependent variable of interest (i.e., prognosis or response to treatment) or phenotype (i.e., somatic mutations, gene expression, protein levels, etc.), and v) testing and validation of the model using independent datasets[8–11]. Notably, in handcrafted radiomics, tumour segmentation determines the region that will be analysed in downstream processes, where semi-automatic and automated methods have been shown to be superior to manual segmentation in NSCLC[9,10,12,13].

Handcrafted Radiomics

Handcrafted radiomic features are broadly categorized into four feature groups: i) tumour intensity (i.e., histogram based), ii) shape, iii) texture based, and iv) filter-based (i.e., wavelet and Laplacian of Gaussian) [10,14–16]. Tumour intensity is calculated using first-order statistics based on the intensity values of single voxels (e.g., maximum, median, minimum, and entropy)[see 16 for review]. Shape is derived from the 3D geometric properties of the VOI and describes features such as volume, surface area, and compactness. Textural (i.e., spatial) features—such as those associated with tumour heterogeneity—are calculated using both second-order statistics derived from the relationship between two voxels (e.g., grey-level occurrence and grey-level run length matrices) and higher-order statistics, which describe the relationship between three or more voxels (e.g., neighbourhood grey-tone difference matrices). Radiomic analyses can also provide information on textures extracted from wavelet-filtered images to assess frequency ranges within the tumour volume and fractals (i.e., model-based features)[9,10,14,16,17].

Delta Radiomics (DelRadX)

Radiomics features have been shown to change significantly over time, most notably in response to treatment[18]. Delta radiomics (DelRadX)—a branch of radiomics that is used to evaluate changes in radiomic features over time (before and after treatment or continuously)—has been used extensively in multiple cancers to capture temporal changes in tumour features such as texture, shape, and compactness, mainly in response to therapy[19]. The use of DelRadX features has also been used extensively in lung cancer to increase the prognostic potential of radiomic features for overall survival[18,20], in combination with conventional radiomics to improve predictions of lung module malignancy[21,22], and to predict response to immunotherapy[23].

Deep Radiomics

In contrast to radiomics, in which predictions are limited to a small number of predetermined features in a relatively simple statistical model, DL models—trained on clinical outcomes—consist of multi-layered architectures that "learn" feature representations in the data in a non-linear, iterative process. This process aims to minimize errors of predictions, in which the outputs of the final layer are considered to be DL features[8,10].

Deep convolutional neural networks (CNNs), such as ResNet[24], have been used extensively in image recognition, medical image categorization, and computer-aided diagnostics[25,26] and are on par with or have surpassed the performance of human experts in numerous image-based diagnostic tasks[4,8]. Deep CNNs can adaptively learn spatial connections between features (i.e., complex visual patterns) without prior definition by human experts, and generally use fully connected layers to transform image representations into a vector of predictions with minimal processing by creating feature maps in convolutional layers using weighted motifs[4,8,27]. Subsequent layers are created using local conjunctions of features detected from previous layers, semantically similar features are merged in several pooling layers, and the fully connected layers translate CNN-extracted DL features into a target output prediction (e.g., treatment response, prognosis, classification of tumours, subtyping, physiological endpoints, somatic mutation)[8,10]. Fully convolutional neural networks—composed only of layers that yield image-like outputs that can be mapped to an image or to delineate a tumour volume—are the most commonly used models for segmentation in medical imaging (e.g., U-Net[28] and V-Net[29])[8,10]. Lastly, fully connected networks—in which all the nodes in one layer are connected to all nodes in the subsequent layer—are suitable for making predictions from non-image data inputs such as radiomic features and clinical variables[8].

While DL modalities are automated, and thus not affected by intra- and inter-observer variability[4,30], DL algorithms generally require a larger dataset than handcrafted radiomics to avoid overfitting the model to the training data[8,9]. Furthermore, in the absence of expert annotations as ground truth labels (i.e., supervised learning), architectures such as deep autoencoders can be used for unsupervised learning tasks with unlabelled data[4,25,26,31,32]. Lastly, a technique called transfer learning (i.e., fine-tuning pre-trained CNN models from natural image datasets for medical image tasks) can be used when data are limited[4,25].

Notably, transfer learning with CNNs that have been extensively trained on non-medical datasets, such as those found on ImageNet, show promising results for automated medical image analysis in a variety of fields [33]. Morid et al. [33] found that DenseNet[34] was the most common model used in lung studies and was successfully used by Wang et al.[35], who used transfer learning to train the first 20 layers of their model in DenseNet, pretrained on 1.28 million images from ImageNet[36], and subsequently trained the last four convolutional layers using 14,926 CT images (with selected ROIs) from lung adenocarcinoma tumours. This DL model, effectively predicted the probability that tumours were epithelial growth factor receptor (*EGFR*) mutants and was statistically superior to hand-crafted methods[37]. Additionally, Ardila et al. [38] developed an extremely high fidelity deep-learning based software to detect lung cancer on low-dose CT scans, while Primakov et al.[12] developed a fully automated pipeline using a modified 2D U-Net for the detection and volumetric segmentation of NSCLC. Lastly, Xu et al.[39] used transfer learning of CNNs (a base ResNet CNN trained on over 14 million images in ImageNet with subsequent CNNs for each timepoint) with recurrent neural networks to train a DL model using a time series of CT images in stage III NSCLC patients treated with chemoradiation. These DL models were significantly predictive of survival and clinical endpoints such as progression, distant metastases, and local-regional occurrence.

Model performance was enhanced with each subsequent scan and the model significantly predicted pathologic response in a validation dataset.

Explainable AI (XAI)

As it is often difficult to identify the precise factors that contribute to AI model outputs, AI is considered a "black box" approach to automating predictions, ultimately hampering the adoption of AI models into clinical workflows[8,9,40]. Furthermore, there is likely an inverse relationship between complexity and model performance (i.e., predictive accuracy) and explainability[41]. Thus, explanatory approaches—such as Gradient-weighted Class Activation Mapping (Grad-CAM)[42], (Local Interpretable Model-Agnostic Explanations) LIME [43], and SHapley Additive exPlanations (SHAP)[44]—aim to increase the transparency and explainability of "black box" choices in AI algorithms via both model-specific and model-agnostic post-hoc XAI analyses[40].
